# Different signaling pathways involved in the anti-inflammatory effects of unfractionated heparin on lipopolysaccharide-stimulated human endothelial cells

**DOI:** 10.1186/s12950-020-0238-7

**Published:** 2020-02-10

**Authors:** Xu Li, Lu Li, Yuequan Shi, Sihan Yu, Xiaochun Ma

**Affiliations:** Department of Critical Care Medicine, the First Affiliated Hospital, China Medical University, North Nanjing Street 155, Shenyang, 110001 Liaoning Province People’s Republic of China

**Keywords:** Unfractionated heparin, Sepsis, Endothelial cells, Signaling pathway, Nuclear factor-κB

## Abstract

**Background:**

There is a complex interplay between inflammatory response and coagulation in sepsis. Heparin is used as a recognized anticoagulant and possesses multiple biological properties that possibly affect sepsis. This study aimed to determine the possible signaling pathways involved in the anti-inflammatory effects of unfractionated heparin (UFH) on lipopolysaccharide (LPS)-stimulated human pulmonary microvascular endothelial cells (HPMECs).

**Methods:**

HPMECs were transfected with siRNA targeting IκB-α. Cells were treated with UFH (0.01 U/ml~ 10 U/ml) 15 min before adding LPS (10 μg/ml). We detected the markers of systemic inflammatory response. Release of interleukin (IL)-6, IL-8 were evaluated at 3 h by ELISA and at 1 h by qRT-PCR. After 1 h, nuclear factor-κB (NF-κB) as well as phosphorylated inhibitor κB-α (IκB-α), signal transducer and activator of transcription-3 (STAT3) and ERK1/2, JNK, p38 mitogen-activated protein kinase (MAPK) expressions were evaluated by Western blot. DNA binding was conducted to further prove the activation of NF-κB pathway.

**Results:**

In HPMECs, UFH obviously inhibited LPS-stimulated production of IL-6 and IL-8, especially in 10 U/ml. UFH inhibited LPS-induced phosphorylation of IκB-α, ERK1/2, JNK, p38 MAPK and STAT3. UFH also suppressed LPS-stimulated nuclear translocation of NF-κB. Importantly, transfection with siRNA targeting IκB-α induced more obvious inflammatory response. UFH suppressed cytokines production and phosphorylation of different signaling pathways in IκB-α silencing cells.

**Conclusion:**

These results demonstrate that UFH exerts the anti-inflammatory effects on LPS-stimulated HPMECs by different signaling pathways.

## Introduction

Sepsis is a complicated clinical syndrome in which the innate host immune response interacts with invading pathogen. There is a complex interplay between inflammatory response and coagulation in sepsis. Release of inflammatory mediators activates the coagulation system, which in turn promotes inflammation through multiple ways, leading to multiple organ dysfunction syndrome (MODS) [1]. Endothelial cells are the monolayer of cells that are located on the inner surface of all blood vessels. They play a major role in host responses during sepsis. Normally, the endothelium acts as a barrier between immune cells and infection or inflammation. However, the properties of endothelial cells in large and microvessels are different. Especially microvascular endothelial cells play an important role in the process of sepsis. Exposure to inflammatory mediators causes the endothelium to activate and become pro-coagulatory and pro-inflammatory [2]. And it is becoming more and more clear, that vice versa, factors of the coagulation system can significantly modulate the inflammatory reaction [1, 3]. An amplified response, however, can result in multiple organ dysfunction and even death. Thus, the agents that suppress the activation of both coagulation and inflammation may improve the prognosis in sepsis.

The anticoagulant properties of unfractionated heparin (UFH) are well established [4]. In these years, more and more attention has been paid to the anti-inflammatory and immunomodulatory effects of UFH [5–10]. As a consequence, UFH has the potential to become a new therapeutic drug for specific group of septic patients. However, the precise mechanism of action is not yet fully elucidated. Our previous studies have shown that UFH suppressed LPS-induced inflammatory mediator production in endothelial cells [8–10]. Whereas, the related mechanisms between anti-inflammatory action and organ protection have not been well documented. This study aimed to further assess the effect of UFH on production of inflammatory markers and the involved signaling pathways in Human Pulmonary Microvascular Endothelial Cells (HPMECs).

## Materials and methods

### Endothelial cells culture and treatment

HPMECs, endothelial cell growth supplement (ECGS) and endothelial cell medium (ECM) were from ScienCell Research Laboratories. HPMECs were cultured in ECM supplemented with 1% ECGS, 5% fetal bovine serum (FBS) and 1% penicillin/streptomycin solution. The cells were cultured at 37 °C in 5% CO_2_. Experiments were conducted with cells at passages 3 to 5. Before each experiment, the cells were rested for 1 h in ECM containing 1% ECGS, 1% FBS and 1% penicillin/streptomycin solution. After incubation, endothelial cells were washed with phosphate-buffered saline (PBS) (PH 7.4) for three times to exclude heparin residues. LPS (LPS from *Escherichia coli* strain 0111:B4, Sigma) was used at 10 μg/ml. The cells were either exposed to LPS alone or in combination with different concentrations of UFH (Shanghai NO.1 Biochem-istry & pharmaceutical Co., China) as specified in the text when they reached 90% confluence.

### Cell viability

The cell viability was evaluated by methyl thiazoyltetrazolium (MTT) assay. HPMECs were seeded in 96-well plates at a density of 1–2 × 10^4^ cells/well. Briefly, at the indicated time after the treatment with or without UFH before exposure to LPS for 24 h, the culture supernatant was removed. The cells were washed with PBS and incubated with 200 μl medium containing 20 μl of MTT (1 mg/ml) at 37 °C for 4 h. The medium was then aspirated and 150 μl of dimethyl sulfoxide (DMSO) per well was added for formazan solubilization. The absorbance of converted dye was measured at a wavelength of 490 nm using a microplate reader. The viability of HPMECs in each well was presented as percentage of control cells.

### Transient transfection and RNA interference

Pre-validated siRNA for human IκB-α (accession number sc-29360) and a negative control (accession number sc-44231) were obtained from Santa Cruz Biotechnology (Santa Cruz, CA, USA). HPMECs at a density of 1 × 10^6^ cells were transfected at 70% confluence with a final concentration of 25 nM either IκB-α siRNA or a scramble control using *Trans*IT-TKO transfection reagent (Mirus, Madison, WI) according to the manufacture’s instructions. HPMECs were cultured for 24 h after transfection and then stimulated with LPS in the presence or absence of varying concentrations of UFH for indicated time, or with UFH alone. UFH was added to cells 15 min prior to stimulation with LPS. The efficiency of gene silencing of IκB-α was determined by western blot and immunofluorescence.

### Enzyme-linked immunosorbent assay (ELISA) for IL-6 and IL-8

HPMECs were treated with UFH 15 min and then exposed to LPS for 3 h. The content of IL-6 and IL-8 in the supernatants of HPMECs were collected and assayed by sandwich ELISA kits according to the manufacturer’s instruction. The minimum detection limit of the assay was 2 pg/ml of protein. ELISA kits for IL-6 and IL-8 were obtained from eBiosciences. The absorbance was measured at 450 nm. The levels of IL-6 and IL-8 were generated from a standard curve.

### Real time reverse transcriptase-polymerase chain reaction (RT-PCR)

RT-PCR was used to detect IL-6 and IL-8 mRNA levels. HPMECs were treated 15 min prior to addition of LPS. After 1 h, total cellular mRNA was extracted from 1.5 × 10^6^ cells using RNeasy Mini Kit (Qiagen, Valencia, CA) according to the manufacture’s protocol. The RNA concentrations were determined by the OD_260_ and OD_260/280_ values that were measured with spectrophotometer. Two microgram of total RNA was reverse transcribed to cDNA and reverse transcription was performed at 42 °C for 30 min and followed by incubation at 85 °C for 5 min. For quantitative PCR, the 10 μl reaction mixture contained 1 μ1 of cDNA template, 3 μl of H_2_O, 1 μl of 10′ primer, 5 μl of 2 × Taq PCR Master-mix. DNA samples were analyzed for cDNA of IL-6, IL-8 and GADPH by PCR amplification using specific primers. The primer sequences used for PCR were designed using Primer 5 software. The primers were as follows: for IL-6: sense: 5′- AGG GCT CTT CGG CAA ATG TA − 3′ and anti-sense: 5′- GAA GGA ATG CCC ATT AAC AAC AA − 3′; for IL-8: sense: 5′-ATT TCT GCA GCT CTG TGT GAA GGT GC-3′ and anti-sense: 5′- TTG TGG ATC CTG GCT AGC AGA C-3′; for GADPH: sense: 5′- CGG AGT CAA CGG ATT TGG TC − 3′ and anti-sense: 5′- CGG TGC CAT GGA ATT TGC CA − 3′. The PCR was started at 95 °C for 10 min, 40 cycles of 95 °C for 10 s, followed by 60 °C for 1 min, and 72 °C for 1 min. The housekeeping gene GADPH was used for normalization. Relative quantification values were calculated by the ΔΔCt method.

### Immunofluorescence

HPMECs were treated with UFH 15 min prior to addition of LPS. After 1 h, cells were fixed in PBS containing 2% paraformaldehyde and permeabilized in PBS containing 0.2% bovine serum albumin and 0.1% Triton X-100 for 15 min at 4 °C. Cells were cultured in blocking solution (2 mg/ml human IgG and 0.5% bovine serum albumin) for 30 min at room temperature. Staining was performed in blocking solution with anti-NF-κB or anti-IκB-α (Cell Signaling Technology, Beverly, MA) antibodies followed by secondary antibodies conjugated to phosphatidylethanolamine or FITC, respectively (Alexa 633 or Alexa 488, Molecular Probes, Eugene, OR). Nuclear staining was performed with 50 ng/ml 4′6- diamidino-2-phenylindole (DAPI, Sigma). The movement of fluorescent label for NF-κB was quantified by the percentage of its location.

### Whole-cell protein extraction and Immunoblotting

Cells were treated with UFH 15 min prior to addition of LPS. After 1 h, cells were lysed in ice-cold urea/CHAPS/Tris buffer (8 M urea, 4% CHAPS, 40 mM Tris-HCl containing DTT, 0.1 mM phenylmethylsulfonyl fluoride, 10 μg/ml of protease inhibitors and phosphatase inhibitors) for 15 min on ice with intermittent vortexing, and extracts were then centrifuged for 10 min at 14,000×*g*. The supernatants were used for immunoblotting. Whole proteins in HPMECs were fractioned by 10% sodium dodecyl sulfate-polyacrylamide gel electrophoresis (SDS-PAGE) and transferred onto Immobilon-P (Millipore) polyvinylidene difluoride (PVDF) membranes electrophoretically. Antibodies recognizing phospho-NF-κB p65 (Ser536), phospho-IκB-α (Ser32), phospho-p44/42 MAPK (Erk1/2) (Thr202/ Tyr204), phospho-p38 MAPK (Thr180/Tyr182), phospho-JNK MAPK (Thr183/Tyr185), phospho-signal transducer and activator of transcription-3 (STAT3) (Tyr 705) were added at a 1:1000 dilution (Cell Signaling Technology, Beverly, MA), whereas antibody to IκB-α was added at a 1:2000 dilution (Cell Signaling Technology, Beverly, MA), and antibody to β-actin was added at a 1:5000 dilution (Santa Cruz Biotechnology, CA). The primary antibody was examined by using autoradiographic film with an HRP-conjugated secondary antibody and an ECL chemiluminescent system (Amersham Pharmacia Biotech, Piscataway, NJ). To prove equal protein loading on gels, β-actin was used as a standard. Secondary antibodies linked to horseradish peroxidase (HRP) and ECL were purchased from Amersham Biosciences. The intensity of immunoreactive bands was determined using Image J software (NIH).

### NF-κB DNA binding assay

An ELISA-based NF-κB DNA binding assay was used to detect the activation of the p65 subunit of NF-κB in the nucleus according to the manufacturer’s instruction (Active Motif, Carlsbad, CA). Briefly, a total of 7.5 μg nuclear extract was prepared using the Nuclear Extraction Kit (Active Motif). Nuclear extracts containing equal amounts of protein were added to the precoated (NF-κB-specific oligonucleotide) 96-well plate. The plate was incubated for 1 h at RT. After three washings of the plate, a primary antibody specific for NF-κB/p65 was added and the plate was incubated for 1 h at RT. After washing three times to remove excess primary antibody, an HRP conjugated secondary antibody was added to each well and incubated for 1 h. Plates were read at 450 nm after addition of the developing reagent.

### Statistical analysis

Data analysis was evaluated by using SPSS 18.0 software. Data were described as mean ± SD from at least three replicates. The results were analyzed by ANOVA with multiple comparisons, and then by student’s paired t-test. The *p*-values lower than 0.05 were considered statistically significant.

## Results

### Effect of UFH on LPS-induced HPMECs injury

**Fig. 1:**
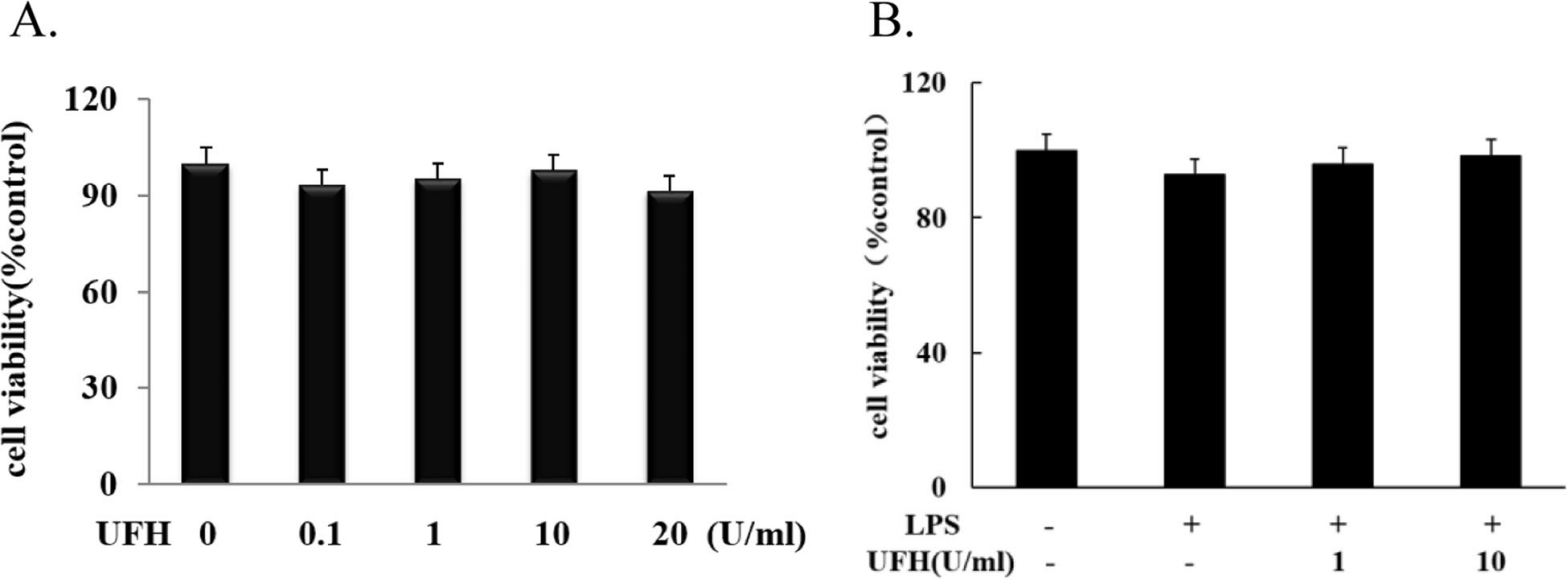
Effect of UFH on cell viability measured by MTT asssay. **a** Cells were treated with 0.1% DMSO or UFH for 24 h. **b** Cells were pre-treated with UFH (1 and 10 U/ml) for 15 min and then exposed to 10 μg/ml of LPS for 24 h. Values are means ± SD of three independent experiments

As cell viability is the most direct indicator to show the cell state, the effects of UFH on viability of LPS-stimulated HPMECs were evaluated. No difference was seen in cell viability between cells treated with UFH (0.1–20 U/ml) alone and controls (Fig. 1a). The results showed that UFH less than the concentration (0.1–20 U/ml) had no detrimental effect on cells. Pre-treatment of HPMECs with various concentrations of UFH (1–10 U/ml) markedly increased the viability of LPS-stimulated HPMECs (Fig. 1b).

### Transfection

**Fig. 2:**
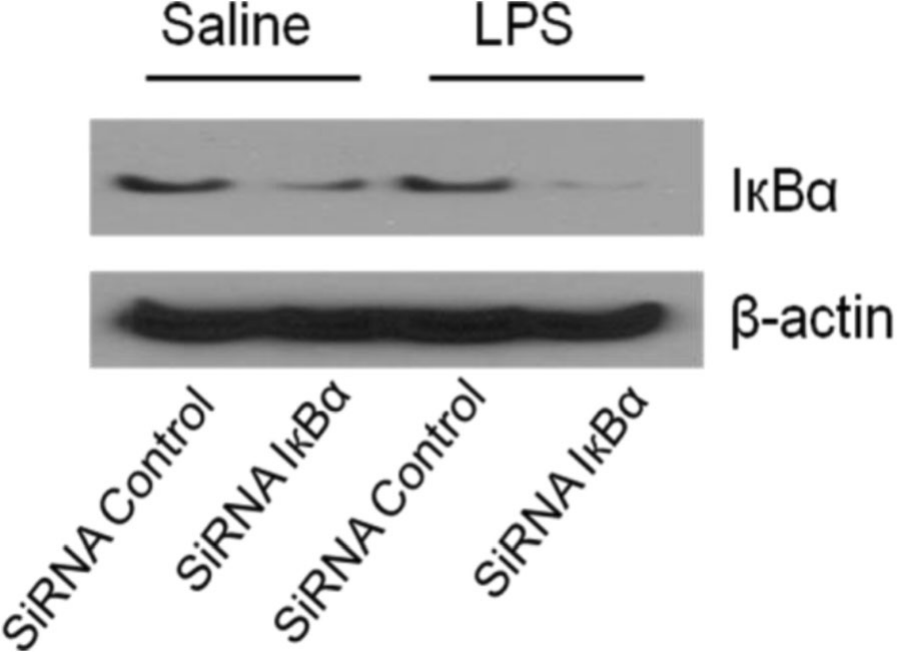
Western blot to prove successful transfection. The results are representative of three independent experiments

**Fig. 3:**
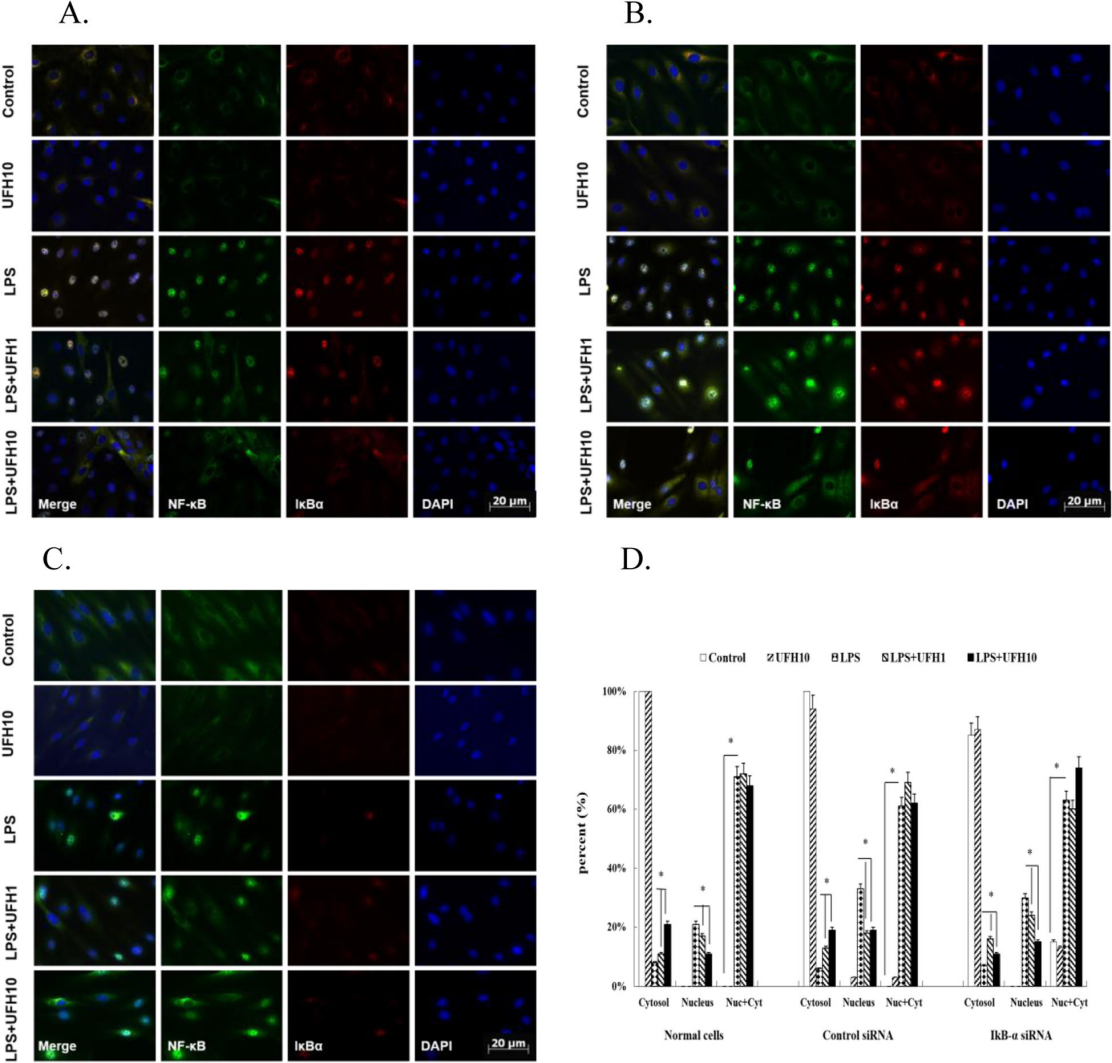
Effects of UFH on nuclear translocation of LPS-stimulated NF-κB p65 by immunofluorescence. Cells were treated with the indicated concentrations of UFH for 15 min, followed by exposure to 10 μg/ml LPS for 1 h. The results are representative of three independent experiments. **a** Untreated cells. **b** Control siRNA cells. **c** IκB-α siRNA cells. **d** Quantification of the movement of fluorescent label for NF-κB. ^*^P<0.05, compared to the vehicle-treated control group. ^**^P<0.01, compared to the vehicle-treated control group. ^#^*P*<0.05, compared to the LPS-treated group. ^##^*P*<0.01, compared to the LPS-treated group

In order to further prove the involvement of NF-κB pathway. We conducted transfection to silence IκB-α. We performed western blot (Fig. 2) and immuno fluorescence (Fig. 3) to prove successful transfection.

### UFH inhibits LPS-induced nuclear translocation of NF-κB by immunofluorescence

Since NF-κB is a vital element participating in the inflammatory response, we further proved the involvement of NF-κB for the pro-inflammatory mediator suppression in LPS-induced HPMECs injury. We determined the effects of UFH on the nuclear translocation of NF-κB p65 subunit. NF-κB existed mostly in the cytoplasm in untreated cells, LPS at the concentration of 10 μg/ml strongly enhanced the nuclear translocation of NF-κB. Remarkably, pre-treatment with UFH retained the NF-κB in the cytosol even when addition of LPS (Fig. 3a). UFH also inhibited activation of NF-κB in IкBα-silencing cells (Fig. 3c). Taken together, the results show that UFH possibly has an anti-inflammatory effect in part by interfering with NF-κB signaling pathway.

### Effect of UFH on IL-6 and IL-8 production induced by LPS

**Fig. 4:**
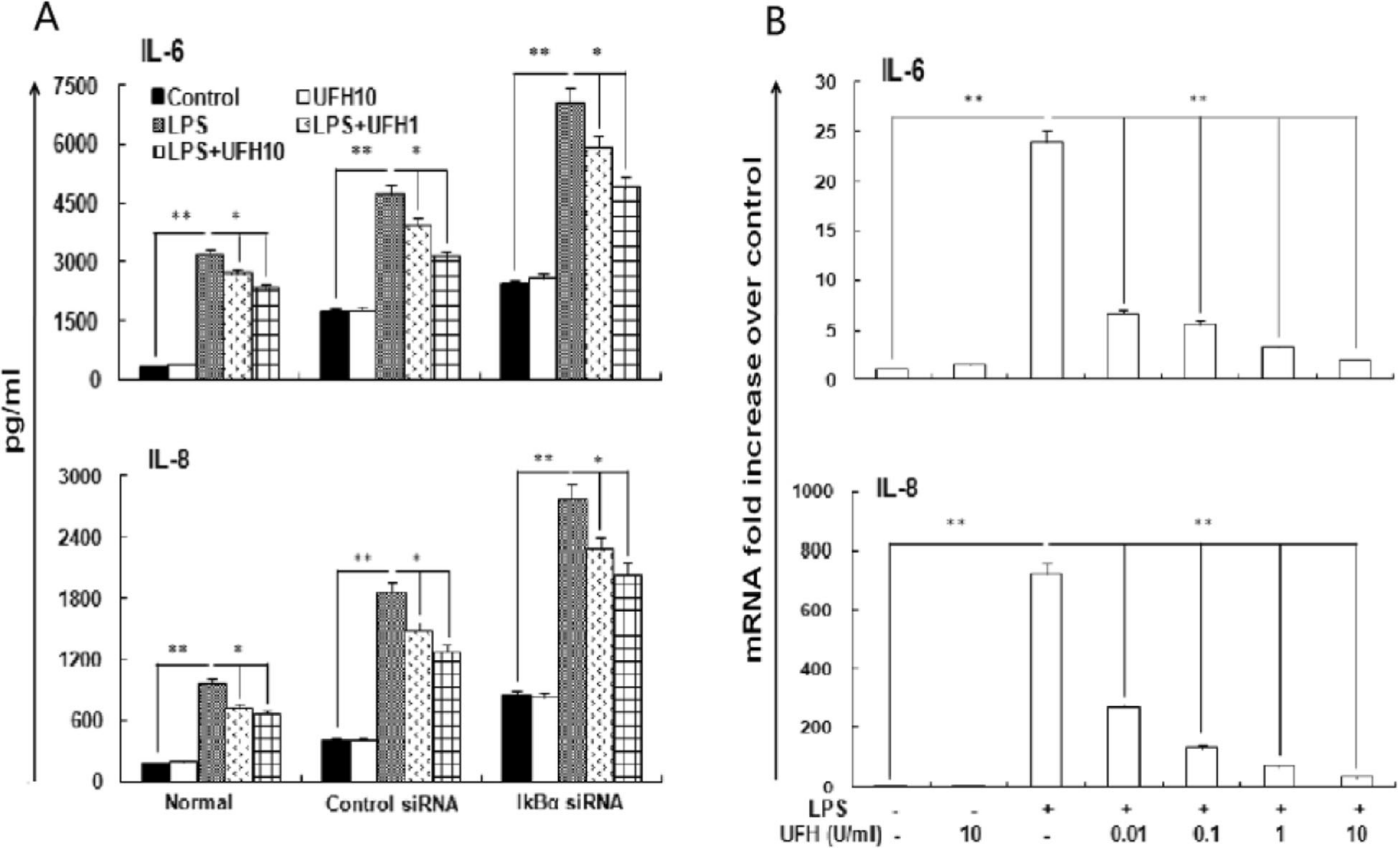
Effects of UFH on the production of IL-6 and IL-8 stimulated by LPS. Supernatants and cells for evaluation of IL-6 and IL-8 were collected at indicated time. UFH suppressed the increasing effects of LPS both on protein (Fig. 4a) and mRNA levels (Fig. 4b). The results represent mean ± SD of three replicates.^*^P<0.05, compared to the vehicle-treated control group. ^**^P<0.01, compared to the vehicle-treated control group. ^#^*P*<0.05, compared to the LPS-treated group. ^##^*P*<0.01, compared to the LPS-treated group

The effect of UFH on LPS-stimulated pro-inflammatory cytokines release was measured at 3 h after LPS stimulation. LPS (10 μg/ml) raised the levels of IL-6 and IL-8 in supernatants. As expected, more inflammatory mediators were released in IкBα-silencing group. UFH decreased LPS-stimulated IL-6 and IL-8 expressions especially in the 10 U/ml group (Fig. 4a).

### UFH depresses LPS-induced IL-6 and IL-8 mRNA expressions

Expressed mRNA levels of IL-6 and IL-8 in HPMECs from three independent experiments were evaluated by real-time PCR. Productions of IL-6 and IL-8 were found to be low in control group, obviously elevated after LPS stimulation. Increased expressions by LPS were significantly decreased by UFH pre-treatment, particularly at the concentration of 10 U/ml (Fig. 4b).

### Effects of UFH on LPS-induced MAPK activation

**Fig. 5:**
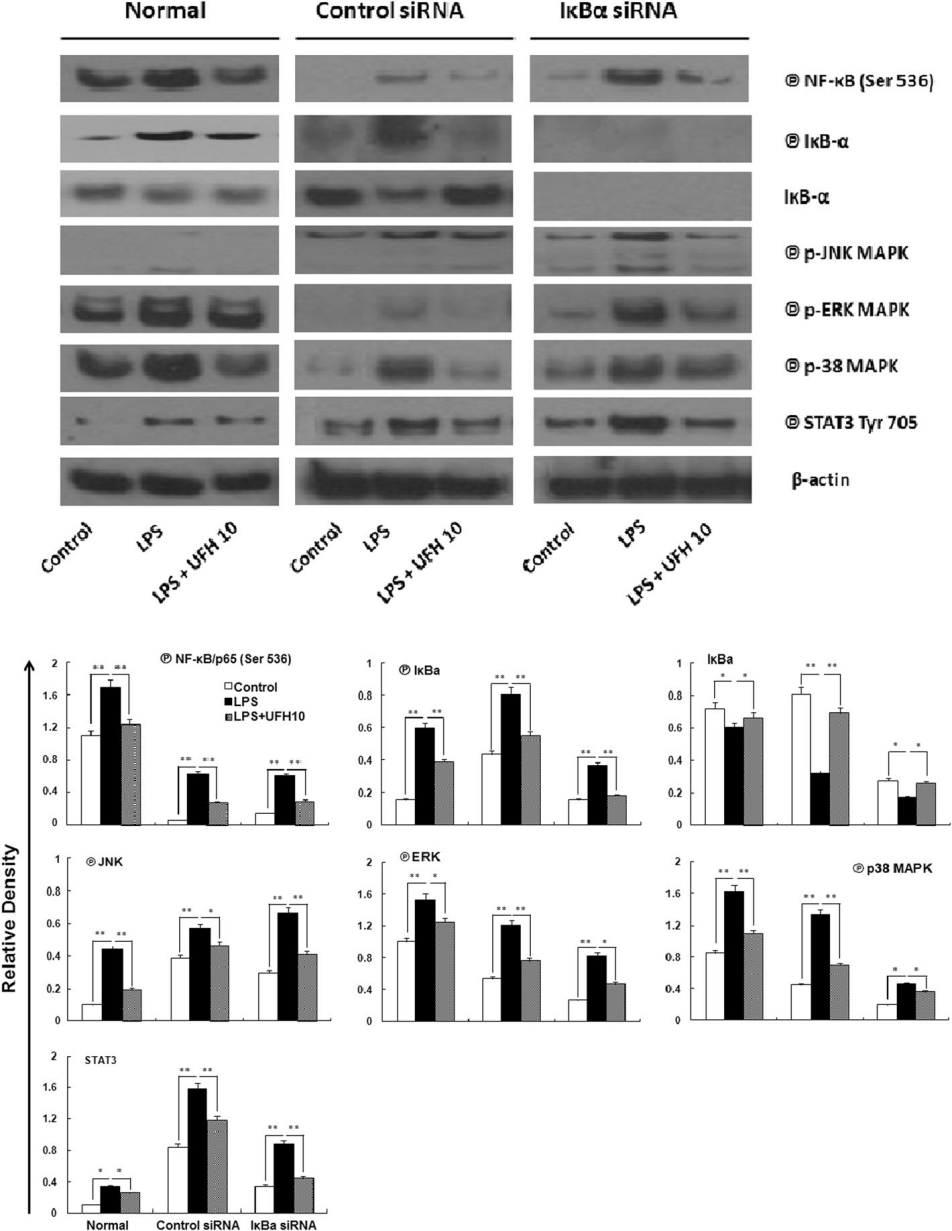
Effects of UFH on LPS-stimulated activation of different signaling pathways. Cells were treated with 10 U/ml of UFH for 15 min, followed by exposure to 10 μg/ml LPS for 1 h. The intensity of the band was corrected with that of β-actin. Graph shows mean ± SD fold change over control from 3 experiments. ^*^P<0.05, compared to the vehicle-treated control group. ^**^P<0.01, compared to the vehicle-treated control group. ^#^*P*<0.05, compared to the LPS-treated group. ^##^*P*<0.01, compared to the LPS-treated group

We detected the possible mechanisms for IL-6 and IL-8 suppression in LPS-induced HPMECs injury by evaluating the effects of UFH on several signaling pathways. The cells were treated with UFH (10 U/ml) for 15 min before addition of LPS (10 μg/ml) for 1 h. As shown in Fig. 5, LPS induced phosphorylation of p38、ERK1/2 and JNK MAPK. UFH treatment blocked MAPK activation in LPS-stimulated HPMECs. UFH also inhibited MAPK activation in IкB-α-silencing cells.

### UFH inhibits LPS-induced activation of NF-κB through inhibition of the degradation of IκB-α

MAPKs play important roles in the activation of NF-κB. We further investigated the effect of UFH on the nuclear translocation of NF-κB by western blot. IκB-α-silencing induced more obvious activation of NF-κB. LPS significantly increased the nuclear translocation of NF-κB. Pre-treatment of UFH retained the NF-κB in the cytoplasm. We next examined the effect of UFH on degradation of IκB-α in LPS-stimulated HPMECs, UFH notably inhibited degradation of IκB-α (Fig. 5). UFH also inhibited activation of NF-κB in IкBα-silencing cells. These results further showed that UFH exerts an anti-inflammatory effect by interfering with the NF-κB-mediated pathway.

### Effect of UFH on LPS-induced STAT3 activation

There may be different transcription factors involved in cytokine expression besides NF-κB. Here we examined the effect of UFH on LPS-induced STAT3 activation in HPMECs. As shown in Fig. 5, STAT3 activation by LPS was also inhibited by UFH in the concentration of 10 U/ml in both normal and IкB-α-silencing cells. These results suggest that UFH exerts protective effect on LPS-induced inflammatory response in HPMECs through different pathways.

### UFH inhibits LPS-induced NF-κB DNA binding activity

**Fig. 6:**
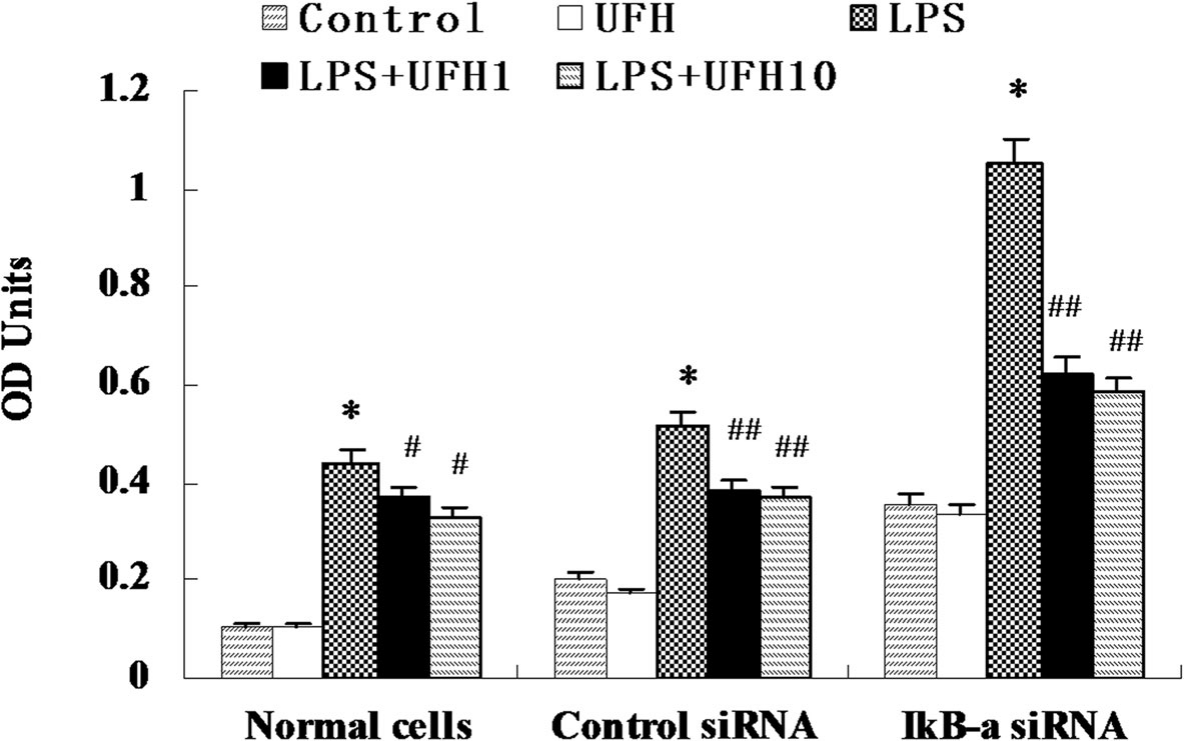
Effects of UFH on LPS-induced NF-κB DNA binding. Cells were pretreated with 10 U/ml of UFH for 15 min, followed by exposure to 10 μg/ml LPS for 1 h. The results are representative of three independent experiments. ^*^P<0.05, compared to the vehicle-treated control group. ^**^P<0.01, compared to the vehicle-treated control group. ^#^*P*<0.05, compared to the LPS-treated group. ^##^*P*<0.01, compared to the LPS-treated group

Actually, activation of NF-κB does not necessarily mean that NF-κB has transcriptional activity. We then performed DNA binding to examine the involvement of NF-κB. As a result, UFH obviously inhibited NF-κB DNA binding activity (Fig. 6).

## Discussion

A continuous barrier of vascular endothelium lined in the inner surface of quiescent blood vessels [11]. Normally, endothelium plays an anticoagulant effect to maintain the homeostasis of the blood system. After being invaded by pathogen, it changes into procoagulant surface to promote the formation of microthrombi [12]. Furthermore, endothelial cells are involved in the process of sepsis as inflammatory cells [13]. When bacteria invade the body, systemic inflammatory reaction leads to cytokine production and endothelial cells activation and injury [14]. The activated endothelium undergoes structural and functional changes, which cause leakage of intravascular components, interaction of leukocytes and endothelial cells and coagulation activation [2]. Thus, endothelial cells acts as first-line responders against invading pathogens in the progress of sepsis [15].

LPS-induced injury of endothelial cells leads to the initiation of proximal signaling events, which lead to the enhanced transcription and expression of pro-inflammatory mediator messenger RNA [16]. Our previous report has shown that UFH has anti-inflammatory properties on LPS-induced HPMECs injury possibly through NF-κB pathway [8–10]. However, the underlying mechanisms to explain the anti-inflammatory effect of UFH remain to be elucidated. Therefore, in this study we exposed the IκB-α-silencing HPMECs to LPS. In vitro, UFH reduced the production of IL-6 and IL-8. Moreover, UFH inhibited LPS-induced NF-κB activation, including degradation of IκB-α and nuclear translocation of p65 even in IκB-α-silencing HPMECs. Our findings further proved the involvement of NF-κB signaling pathway in the protective effects of UFH on LPS-stimulated endothelial cells as our previous reports. These results are also consistent with in vivo experiment which showed the anti-inflammatory effect of UFH via inactivation of NF-κB pathway [17–19].

Though NF-κB commonly participates in the transcriptional up-regulation of pro-inflammatory mediators, some experiments indicate that increases in cytokine production may be also caused by different transcription factors, such as MAPK, STAT3, AP-1 [20]. Here we demonstrated that pre-incubation with UFH blocked the LPS-induced phosphorylation of STAT3 in both normal and IκB-α-silencing cells. And pre-incubation with UFH significantly inhibited LPS-stimulated MAPK activation by the inhibition of phosphorylated p38、ERK1/2 and JNK levels in both normal and IκB-α-silencing cells. These hallmark features totally offer proof for the involvement of MAPK, NF-κB and STAT3 signaling pathway in HPMECs stimulated by LPS. Hence, the modulation of these events by UFH explains its protective effect against LPS-stimulated expression and release of pro-inflammatory mediators. Thus, based on present data, it is probably that UFH exerts its anti-inflammatory effects on LPS-induced HPMECs injury by inhibiting the activation of receptor-mediated signaling pathways, involving MAPK、NF-κB and STAT3. Further research is needed to find out which is the main pathway.

As for the down-regulation of the mentioned pathway in our experiments could be explained by interference with TLR4 ligand binding. Anastase-Ravion et al. [21] had shown that heparin inhibits the binding of LPS to cells via a CD14-independent pathway. We observed LPS-RS (an antagonist for TLR4) inhibited LPS-induced up-regulation of IL-6 and G-CSF. UFH might take its therapeutic effect through TLR4-dependent pathway [22].

## Conclusion

In this study, we further proved the molecular mechanisms involved in the anti-inflammatory effects of UFH on LPS-induced HPMECs injury. These protective responses seem to be mediated by blocking of the pro-inflammatory MAPK、NF-κB、STAT3 signaling pathways. In terms of clinical relevance, these data collectively suggest that UFH might be a promising agent for the therapy of sepsis.

## Data Availability

The data used to support the findings of this study are available from the corresponding author upon request.

## Abbreviations

DMSO: Dimethyl sulfoxide
ECGS: Endothelial cell growth supplement
ECM: Endothelial cell medium
ELISA: Enzyme-linked immunosorbent assay
FBS: Fetal bovine serum
HPMEC: Human pulmonary microvascular endothelial cells
IL: Interleukin
IκB-α: Inhibitor κB-α
LPS: Lipopolysaccharide
MAPK: Mitogen-activated protein kinase
MODS: Multiple organ dysfunction syndrome
MTT: Methyl thiazoyltetrazolium
NF-κB: Nuclear factor-κB
PBS: Phosphate-buffered saline
RT-PCR: Real time reverse transcriptase-polymerase chain reaction
STAT3: Signal transducer and activator of transcription-3
UFH: Unfractionated heparin
